# Genetic diversity and population structure of domestic and wild reindeer (*Rangifer tarandus* L. 1758): A novel approach using BovineHD BeadChip

**DOI:** 10.1371/journal.pone.0207944

**Published:** 2018-11-30

**Authors:** Veronika Ruslanovna Kharzinova, Arsen Vladimirovich Dotsev, Tatiana Evgenievna Deniskova, Anastasiya Dmitrievna Solovieva, Valeriy Ivanovich Fedorov, Kasim Anverovich Layshev, Tatiana Michailovna Romanenko, Innokentiy Michailovich Okhlopkov, Klaus Wimmers, Henry Reyer, Gottfried Brem, Natalia Anatolievna Zinovieva

**Affiliations:** 1 L.K. Ernst Federal Science Center for Animal Husbandry, Federal Agency of Scientific Organizations, pos. Dubrovitsy, Podolsk Region, Moscow Province, Russia; 2 Federal Government Budget Scientific Institutions Yakut Scientific Research Institute of the Agriculture Federal Agency Scientific Institutions, Yakutsk, Russia; 3 North-West Center of Interdisciplinary Researches of Food Maintenance Problems, Federal Agency of Scientific Organizations, St. Petersburg, Russia; 4 Federal Center for Integrated Arctic Research [FCIARctic] Nenets Division—Agro-Experimental Station, Federal Agency of Scientific Organizations, Naryan-Mar, Nenets AO, Russia; 5 Institute for Biological Problems of Cryolithozone Siberian Branch of RAS, Yakutsk, Russia; 6 Institute of Genome Biology, Leibniz Institute for Farm Animal Biology [FBN], Dummerstorf, Germany; 7 Institute of Animal Breeding and Genetics, University of Veterinary Medicine [VMU], Vienna, Austria; Kunming Institute of Zoology, Chinese Academy of Sciences, CHINA

## Abstract

Reindeer (*Rangifer tarandus* L. 1758) are an essential element of the Russian Far North, providing a significant source of nutrition for the representatives of 18 ethnicities. The species has wild and domestic forms, which are in constant interaction. The aim of our study was to characterize the genetic structure of domestic and wild reindeer populations, using a genome-wide bovine genotyping array (BovineHD BeadChip). The wild reindeer samples were obtained from the western Taymyr Peninsula population and from the taiga and tundra populations in the Sakha Republic (Yakutia). The domestic populations included the Evenk, Even, and Chukotka-Khargin breeds of Yakutia and the Nenets breed from the Nenets Autonomous district and Murmansk region. The level of genetic diversity was higher for the wild population. Analyzing Neighbor-Net tree, multidimensional scaling, and Structure results, we observed strong genetic population structure and clear differentiation between domestic and wild populations. All regional populations of domestic reindeer were clearly separated, while wild reindeer showed similar genetic backgrounds. Nevertheless, we found contrasting patterns in the genetic structure of the tundra and taiga reindeer, in accordance with their morphological and ecological differences. Thus, our study revealed a clear genetic differentiation between domestic and wild reindeer populations. It provides novel insights into the genetic diversity and structure of reindeer populations, to support resource utilization and aid in the development of genetic improvement strategies and conservation programs for this species.

## Introduction

Reindeer (*Rangifer tarandus* L. 1758), also known as caribou in North America, is a deer species with circumpolar distribution and is native to Arctic, Subarctic, tundra, boreal, and mountainous regions of northern Europe, Siberia, and North America [1; 2]. Among modern ruminants, it is the only species having both wild and domestic forms, which are in constant interaction [3; 4].

According to the Arctic Council’s project “Sustainable Reindeer Breeding” [5], Russia has approximately 2/3 of the world’s domestic reindeer stock browsing in tundra, forest tundra, boreal forest (taiga), and mountainous regions covering over 3 million km^2^. Unlike Norway, Sweden, and Finland, Russian reindeer husbandry is much differentiated: representatives of 18 ethnicities are engaged in the industry and preserve their national traditions through reindeer husbandry, and 16 of them are included on the official list of indigenous small-numbered ethnicities of the North [6].

In 1985, by order No. 212 of the Russian Ministry of Agriculture (formerly the USSR Ministry of Agriculture), four reindeer breeds were officially recognized: Nenets, Even, Evenk, and Chukotka [7]. In addition, breed-specific ecotypes exist. For instance, the reindeer of Chukotka origin in the Republic of Sakha (Yakutia) are called Khargin. All reindeer breeds are the result of selection by different northern communities and are characterized by their behavior and adaptability to their respective environments [8].

Besides domestic reindeer, there are many wild reindeer in Russia, whose range comprises nearly the entire tundra, forest-tundra, and taiga zones. However, the main population (about 85%) is concentrated in three large regions: Taymyr Peninsula (Taymyr), northern Yakutia, and central Chukotka [6]. The Taymyr reindeer herd is the largest and most monitored wild reindeer population in Eurasia, inhabiting a vast territory in north central Siberia that spans 1.5 million km^2^ [9]. The wild Yakutia herds include tundra and taiga populations [10]. Additionally, wild Yakutia tundra reindeer are represented by three relatively isolated populations: Leno-Olenek, Yano-Indigir, and Sundrun [11]. The wild taiga population is divided into three relatively isolated spatial groups: west Yakutia, south Yakutia, and mountain-taiga [12]. Along with domestic reindeer, the wild ones are a very important component of aboriginal economies and have significant cultural value [13].

Both wild and domestic reindeer are united into two large ecological forms: tundra and taiga. In domestic reindeer, the tundra forms are Nenets and Chukotka breeds, while the taiga forms are Even and Evenk breeds [14].

In Russia, domestic and wild reindeer population numbers have drastically decreased, likely because of changes in economic priorities, global climate change, and industrial development. The number of domestic reindeer over the last few decades has decreased; as of January 1, 2012, it was assessed at 1,583,000 livestock units, or 70.0% of that in 1990 [15]. The situation with the wild population is more dramatic. The current population of the wild Taymyr herd has declined to between 400,000 and 500,000, which is a stark contrast from one million in 2000 [16]. Currently, the total number of the wild reindeer in Yakutia is estimated at 215,000, including tundra populations of 130,000 and forest populations of 85,000 [10].

For reindeer breeding, the coexistence of domestic and wild populations poses serious problems [17]: 1) domestic reindeer joining wild herds, 2) grazing of pastures and mutual pasture competition, 3) outbreaks of infections, and 4) disease transmission. The first two factors result in a complete lack of domestic reindeer breeding in the central Taymyr, and a substantial reduction in domestic reindeer livestock in eastern Taymyr and some other regions [18]. Recent research (October 2013) on Taymyr herd migration to northwest Yakutia revealed a negative consequence of their presence in this territory: the loss of 1500 domestic reindeer that used to graze in the Malaya and Bol'shaya Kuonamka River basins [19]. According to Davydov [20], following migration, wild bulls joined domestic herds during the rut and mated with cows, which resulted in mixed calves accounting for 3% of total offspring.

Regarding the intraspecific status of domestic reindeer, some authors have hypothesized that in the same geographical areas they form a common gene pool with the wild species, which results in introgression of domestic lineages into the wild gene pool [21–23]. However, recent studies by Anderson et al. [4] showed that the reindeer herders in northeastern Zabaĭkal'e have developed an effective breeding technique that, while mixing pedigrees in the short term, guards against wholesale introgression between wild and domestic populations over the long term. Nevertheless, this study was carried out on wild and domestic reindeer populations whose migrations are limited by mountain range. The issues concerning genetic structure and differentiation of regional populations of domestic and wild reindeer inhabiting northeast Russia have not been previously studied.

To effectively manage reindeer populations and overcome the negative effects of their decline, it is necessary to apply modern approaches for assessing and preserving the biodiversity of this important species [24]. The development of genetic markers for deer has followed the development of biochemistry and molecular biology, from the initial studies that utilized allozymes to studies that incorporate molecular markers derived from both mitochondrial DNA and the nuclear genome [25]. Although microsatellites have been widely applied to investigate genetic diversity and population structure, single nucleotide polymorphism (SNP) markers have gradually replaced them, due to their abundance, cost efficiency, and ease of automation [26]. The emergence of high-throughput SNP genotyping facilities, coupled with the gradual reduction in genotyping costs, may help elucidate the genetic diversity and structure of endangered populations [27]. Despite their attractiveness, there are some difficulties in using SNP in non-model organisms due to limited availability of genomic resources, leading to complex laboratory screening of segments of the genome from multiple individuals to yield few independent SNPs [28]. However, recent studies showed successful application of a commercial DNA chip (designed for domestic animals) for cross-species genotyping, analyzing SNP distribution diversity within groups of animals and genetic distance among studied species [29–34]. The results obtained in our previous studies have shown that medium-density DNA chips developed for cattle can be successfully applied to evaluate the genetic diversity and differentiation of some Russian reindeer populations [23; 34]. Until now, the BovineHD BeadChip genotyping array had never been applied to domestic and wild reindeer.

We used the BovineHD BeadChip for the first time to study the genetic diversity and population structure of regional populations of domestic and wild reindeer inhabiting the Russian Far North, an area extending over 4000 km from the west to the east. We further attempted to determine the differences among four domestic reindeer breeds within Nenets Autonomous district, Murmansk region and Yakutia, as well as among wild tundra and taiga forms inhabiting Yakutia and western Taymyr. Our results provide a scientific basis for maintaining genetic diversity and preventing the loss of this important resource, not only for Russia but the whole Arctic zone.

## Materials and methods

### Ethics statement

This study does not involve any endangered or protected species. All wild reindeer muscle tissue samples were collected during scientific expeditions after obtaining collection permits granted by the Department of Hunting of the Republic of Sakha and Taymyrsky Dolgano-Nenetsky District, in compliance with the Russian Federation Law No. 209-FZ of July 24, 2009. The domestic reindeer tissue samples were collected by trained personnel under strict veterinary rules. Sampling was performed in accordance with the ethical guidelines of the L.K. Ernst Federal Science Center for Animal Husbandry. The protocol was approved by the Commission on the Ethics of Animal Experiments of the L.K. Ernst Federal Science Center for Animal Husbandry (Protocol Number: 2018/1). The biomaterials from the genetic resource collection of the L.K. Ernst Federal Science Center for Animal Husbandry, supported by the Federal Agency for Scientific Organizations, were used in the study.

The genotyping data presented in this article will be available through the Data Dryad digital repository.

### Sample collection, DNA extraction, and genotyping

**Fig 1:**
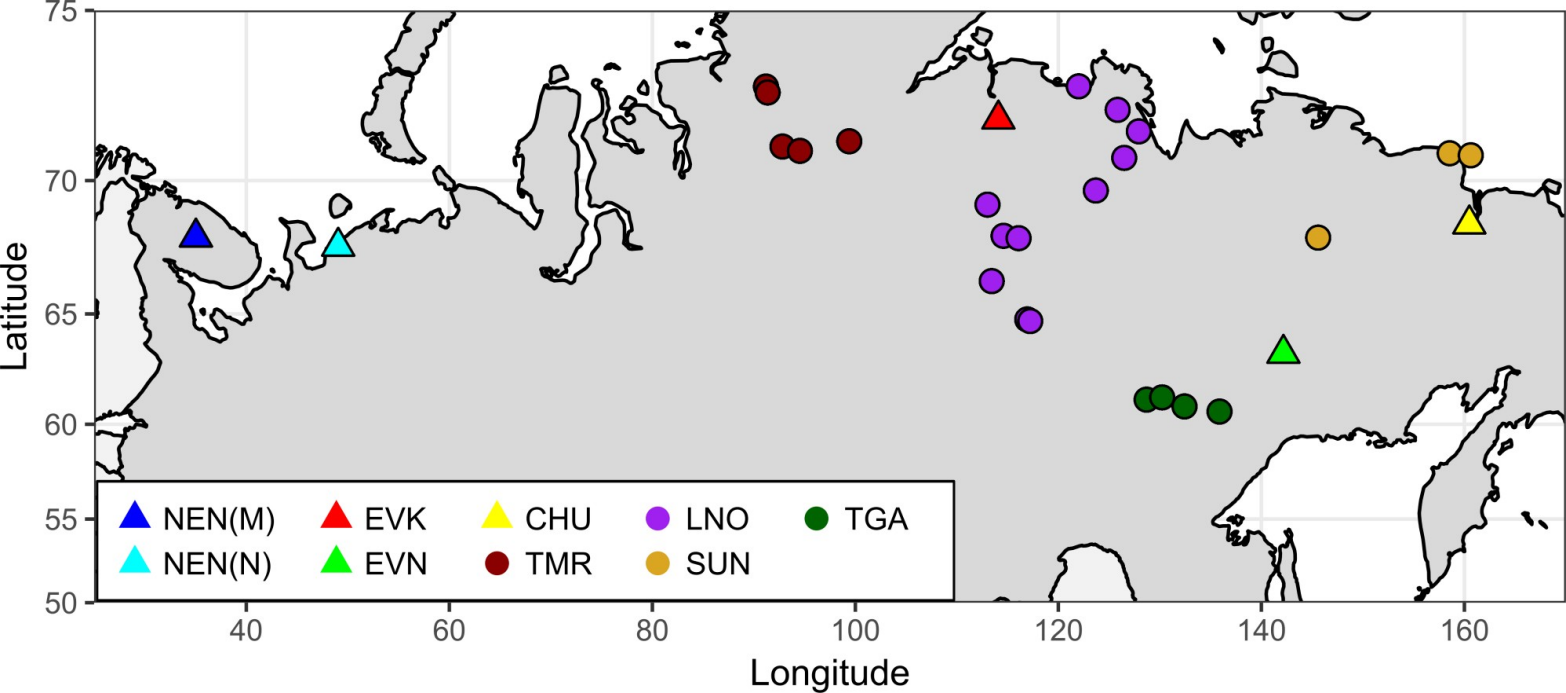
Map of the Russian Far North showing the distribution of the wild and domestic reindeer sampling sites. Note: NEN_M–Nenets breed of the Murmansk region; NEN_N–Nenets breed of the Nenets Autonomous district; EVK–Evenk breed; EVN–Even breed; CHU–Chukotka-Khargin breeds; and TMR–Taymyr Peninsula, LNO–Leno-Olenek, SUN–Sundrun, and TGA–taiga (boreal forest) wild populations.

A total of 135 individuals, including wild (n = 61) and domestic (n = 74) reindeer, were analyzed (Fig 1).

The wild reindeer samples were represented by 27 individuals from the Taymyr population from western Taymyr (TMR) and 34 from the Yakut population from the taiga and tundra of Yakutia. In addition, the wild reindeer from Yakutia included two populations of tundra reindeer from northern Yakutia (Lena-Olenek (LNO, n = 24) and Sundrun (SUN, n = 6)) and one population of taiga reindeer from southern Yakutia (TGA, n = 4). All samples were collected during scientific expeditions between 2014–2017. The coordinate range of the covered area in Taymyr varied from 70° to 74°N and from 91 to 107°E; in Yakutia, it ranged from 59 to72°N and from 113 to 145°E. The geographic map (with longitude and latitude coordinates for each sampling site) was plotted using the R packages maps and mapdata [35].

The domestic reindeer tissue samples of were taken from the Even (EVN, n = 7), Evenk (EVK, n = 12), and Chukotka-Khargin (CHU, n = 2) breeds from three farms in Yakutia (“Yuchyygeyskoe”, Oymyakonsky district; “Reindeer Company named after I. Spiridonov”, Anabarsky district; and “Turvaurgin”, Nizhnekolymskiy district, respectively); and the Nenets breed samples were taken from the Nenets Autonomous district (NEN_N, n = 33, “Indiga”, Malozemelskaya tundra) and the Murmansk region (NEN_M, n = 20, “Tundra”, northern and northeastern Kola Peninsula). The biomaterial was collected during corral work on the herd throughout 2016–2017.

Genomic DNA was extracted from muscle and tissue samples using Nexttec columns (Nexttec Biotechnology GmbH, Germany) following the manufacturer's instructions. The quality of the extracted DNA was examined by electrophoresis using 1% agarose gels viewed under ultraviolet light. The concentration of DNA solutions was quantified using a Qubit 3.0 fluorimeter (Thermo Fisher Scientific (formerly Life Technologies), Wilmington, DE, USA). The OD260/OD280 ratio of DNA solutions was determined by NanoDrop-2000 (Thermo Fisher Scientific, Wilmington, DE, USA).

All reindeer individuals were genotyped with Illumina BovineHD Genotyping BeadChip, which contains 777,962 SNPs. Genotypes were called and processed using Genome Studio (Illumina, Inc. San Diego. USA). Samples with a call rate below 90% were excluded from the data set. Additional criteria to filter SNPs were applied: SNPs with more than 10% missing genotypes across all the samples; SNPs with minor allele frequency less than 5% (—maf 0.05); SNPs located on sex chromosomes of the UMD 3.1 assembly [36], as well as SNPs with unknown map positions; SNPs with the value of linkage disequilibrium (LD) between a pair of single nucleotide polymorphisms equal to r^2^>0.05 (we used a sliding window of 50 SNPs, sliding along in 5 SNP increments); and SNPs not corresponding to the χ2 criterion for Hardy-Weinberg equilibrium in a population (p≤1×10^−6^). Additionally, to assess the quality of SNP genotyping, we used GC Score (quality of reading SNP) and GT Score (level of clustering SNP) of at least 0.5 (50%). These quality control steps were carried out using PLINK v1.07 [37]. The final data set comprised 8357 SNPs for 135 reindeer individuals and 8145 SNPs for 61 wild reindeer from the BovineHD BeadChip (, Illumina, Inc. San Diego. USA).

### Genetic diversity and differentiation analysis

To assess genetic diversity of the studied reindeer populations, the values of observed (*H*o) and unbiased expected (*H*e) heterozygosity, inbreeding coefficient (*F*_IS_) [38], and rarified allelic richness (*A*r) were calculated in R package diveRsity [39]. We collected too few Chukotka-Khargin breed samples to be included in the diversity estimation, though they were considered in other analyses.

Pairwise fixation index (*F*_ST_) values [40] were estimated through the R-package StAMMP [41]

The neighbor-joining algorithm [42] was applied to generate the Neighbor-Net Tree from a distance matrix of pairwise *F*_ST_ values and implemented in Splitstree 4.14.5 [43].

Multidimensional scaling (MDS), based on pairwise identity-by-state distance matrix, was carried out with PLINK 1.07 (—cluster,—mds-plot 4) and visualized in R package ggplot2 [44]. This analysis was performed for all studied populations based on 8357 SNPs, as well as for the wild population based on 8145 SNPs.

### Genetic structure analysis

The genetic structure analysis was performed with the software package STRUCTURE 2.3.4 [45]. Admixture was allowed with a single value of delta K (ΔK) inferred for all populations. We ran 100 simulations for each K value (the number of assumed populations) from one to seven using a burn-in of 10,000 and 100,000 Markov chain Monte Carlo (MCMC) for each value of K. We used STRUCTURE HARVESTER [46] with the Evanno method [47] to determine the most adjustable ancestral populations that would best fit the current data.

### TreeMix

For inferring the patterns of population splits and gene flow between reindeer populations, we used the software TreeMix1.13 [48]. The wild taiga population (TGA) was set as a root out-group. First, a maximum likelihood tree of the reindeer populations was constructed without migration events. Then, migration events were added to the tree one at a time to obtain the most frequently found variants with significant gene flow. Standard errors (-se) and p-values were calculated with jackknife blocks of 10 SNPs (-k 10). Since significant effects were revealed when two migration events were allowed (p<0.05), we ran 100 independent replicates for each event. The tree graph and residuals were visualized using R. In addition, we calculated the *f*_3_ statistic (with -k 10 over a set of 8357 SNPs) using the software threepop within TreeMix [49].

## Results

### Genetic diversity and differentiation analysis

The analysis of genetic variability parameters including observed (*H*o), unbiased expected (*H*e) heterozygosity, rarified allelic richness (*A*r), and inbreeding coefficient (*F*_IS_) for the studied reindeer groups is presented in Table 1.

**Table 1:** Parameters of genetic diversity of domestic and wild reindeer populations based on 8357 SNPs.

Breed or Population	n	*H*o (±se]	*H*e (±se]	*A*r (±se]	*F*_IS_ (*F*_IS_ 95% CI > 0)
Domestic reindeer					
NEN_M	20	0.168±0.002	0.172±0.002	1.314±0.003	0.019 [0.014; 0.024]
NEN_N	33	0.166±0.002	0.167±0.002	1.305±0.003	0.008 [0.003; 0.012]
EVK	12	0.172±0.002	0.175±0.002	1.319±0.004	0.014 [0.007; 0.020]
EVN	7	0.167±0.002	0.169±0.002	1.308±0.004	0.014 [0.005; 0.023]
In total	74	0.167±0.002	0.175±0.002	1.320±0.003	0.044 [0.040; 0.047]
Wild reindeer					
TMR	27	0.172±0.002	0.175±0.002	1.320±0.003	0.013 [0.009; 0.017]
LNO	24	0.176±0.002	0.178±0.002	1.327±0.003	0.012 [0.008; 0.017]
SUN	6	0.172±0.002	0.174±0.002	1.319±0.004	0.010 [0.001; 0.018]
TGA	4	0.153±0.002	0.173±0.002	1.315±0.004	0.089 [0.077; 0.101]
In total	61	0.172±0.002	0.177±0.002	1.325±0.003	0.026 [0.022; 0.029]

Note: NEN_M–Nenets breed of the Murmansk region; NEN_N–Nenets breed of the Nenets Autonomous district; EVK–Evenk breed, EVN–Even breed; and TMR–Taymyr Peninsula, LNO–Leno-Olenek, SUN–Sundrun, and TGA–taiga (boreal forest) wild populations; *H*o–observed heterozygosity, *H*e–expected heterozygosity, *А*r–rarified allelic richness; and *F*_IS_−coefficient of inbreeding.

Genetic diversity was higher for the wild population (*H*o = 0.172, *H*e = 0.177), compared to the domestic breeds (*H*o = 0.167, *H*e = 0.175). Meanwhile, allelic richness was similar for both populations, ranging from 1.305 in NEN_M to 1.327 in LNO. All reindeer groups showed heterozygous deficiency (*F*_IS_ = 95%, CI>0), as evidenced by the slightly positive average inbreeding index, with higher values in domestic breeds (*F*_IS_ = 0.044). Among domestic reindeer, the highest average observed heterozygosity was detected in EVK (*H*o = 0.172), while others were characterized by similar values (*H*o = 0.168, 0.166, and 0.167 for NEN_M, NEN-N, and EVN, respectively). Regarding the average expected heterozygosity, its prevalence was observed in two breeds: NEN_M (*H*e = 0.172) and EVK (*H*e = 0.175). The Lena-Olenek wild population showed the highest degree of genetic diversity (*H*o = 0.176, *H*e = 0.178, *A*r = 1.327), while the taiga reindeer exhibited extremely low genetic diversity (*H*o = 0.153, *A*r = 1.315). Inbreeding level was also high within the taiga population (*F*_IS_ = 0.089).

Pairwise *F*_ST_ values and genetic distances, based on 8357 SNPs, are given in Table 2.

**Table 2:** Genetic differentiation of domestic and wild reindeer populations measured by *F*_ST_ values.

Breed or Population	Domestic reindeer					Wild reindeer			
	NEN_M	NEN_N	EVK	EVN	CHU	TMR	LNO	SUN	TGA
NEN_M	0.000								
NEN_N	0.018	0.000							
EVK	0.039	0.046	0.000						
EVN	0.062	0.075	0.032	0.000					
CHU	0.070	0.086	0.042	0.054	0.000				
TMR	0.038	0.061	0.049	0.066	0.062	0.000			
LNO	0.040	0.063	0.049	0.064	0.062	0.004	0.000		
SUN	0.044	0.070	0.054	0.072	0.072	0.010	0.009	0.000	
TGA	0.067	0.094	0.072	0.087	0.078	0.034	0.031	0.035	0.000

Note: NEN_M–Nenets breed of the Murmansk region; NEN_N–Nenets breed of the Nenets Autonomous district; EVK–Evenk breed, EVN–Even breed; CHU–Chukotka-Khargin breed; and TMR–Taymyr Peninsula, LNO–Leno-Olenek, SUN–Sundrun, and TGA–taiga (boreal forest) wild populations. All *F*_ST_ values were significantly different from 0 in all pairwise comparisons between all groups (p<0.01).

The pairwise comparison of *F*_ST_ values showed high genetic differentiation between domestic and wild populations. Lower values were observed between NEN_M and two wild populations (TMR and LNO), while NEN_N paired with TGA had the highest *F*_ST_ value (0.094).

Within the domestic population, the lowest differentiation (0.018) was detected between the two Nenets breeds. However, these two populations were genetically more distant from the CHU (CHU/NEN_N = 0.086, CHU/NEN_M = 0.070) and EVN (EVN/NEN_N = 0.075, EVN/NEN_M = 0.062). Notably, low to moderate *F*_ST_ values were observed between Yakutia reindeer breeds, ranging from 0.032 for EVN/EVK to 0.054 for EVN/CHU.

All groups of the wild reindeer had low *F*_ST_ values (≤0.05) and TMR tended to have lower levels of differentiation with LNO (0.004), though the degree of differentiation was greater when comparing TGA with the rest of the populations: TMR, 0.034; LNO, 0.031; and SUN, 0.035.

**Fig 2:**
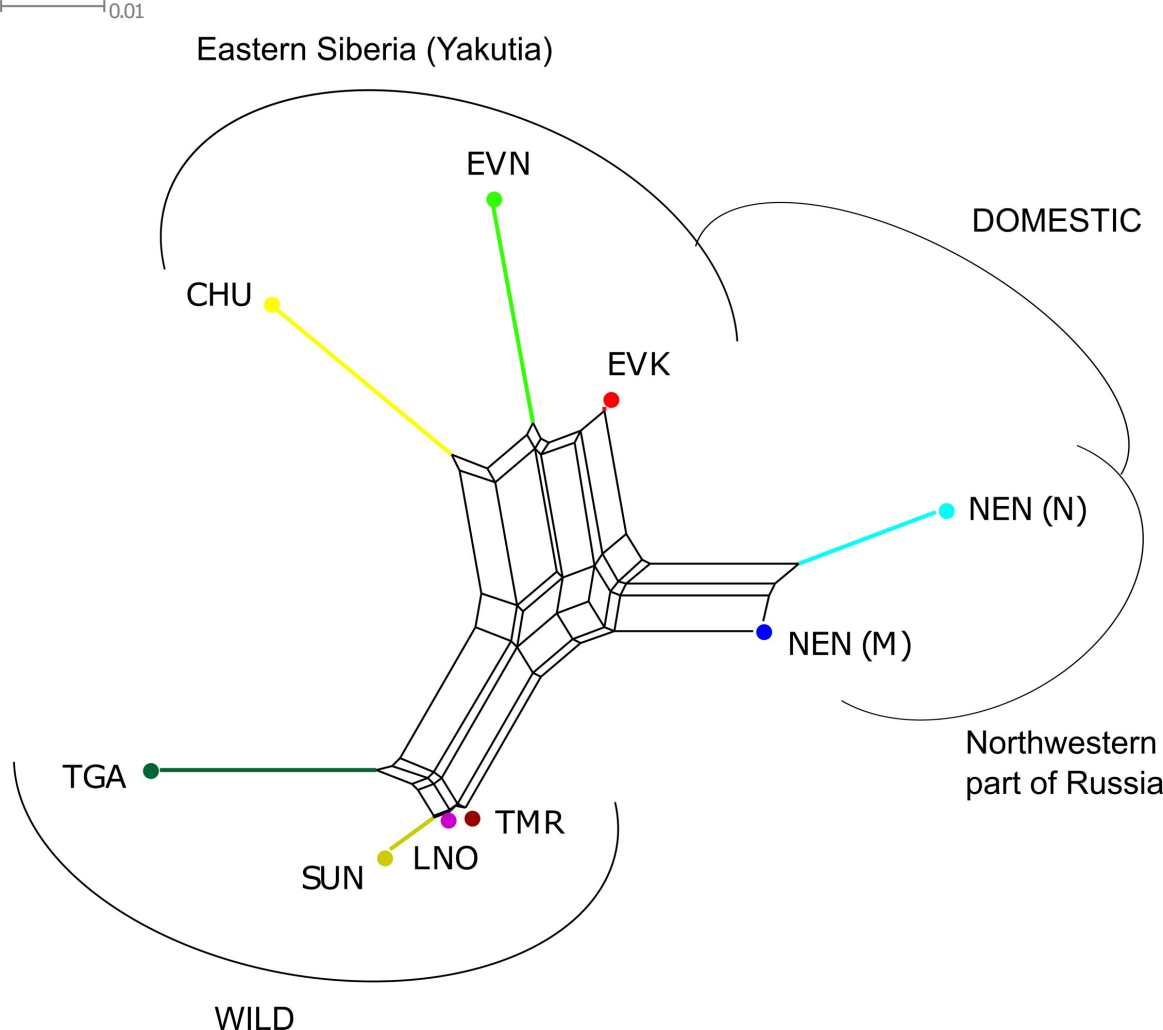
Neighbor-Net tree based on pairwise *F*_*ST*_ values between reindeer populations. Note: NEN_M–Nenets breed of the Murmansk region; NEN_N–Nenets breed of the Nenets Autonomous district; EVK–Evenk breed, EVN–Even breed; CHU–Chukotka-Khargin breed; and TMR–Taymyr Peninsula, LNO–Leno-Olenek, SUN–Sundrun, and TGA–taiga (boreal forest) wild populations.

Regarding the Neighbor-Net tree, which was constructed based on pairwise *F*_*ST*_ values distance matrix (Fig 2), the wild population clearly differed from domestic reindeer, which are kept on farms in the different regions. The populations within Yakutia occupy parallel locations and the Nenets breeds are separated by one branch. The groups of wild reindeer were placed close to the each other: the SUN, TMR, and LNO were found on one large branch of the tree with the lowest divergence between TMR and LNO. However, the TGA reindeer were placed on the greatest distance from them and were positioned outside of the clade.

**Fig 3:**
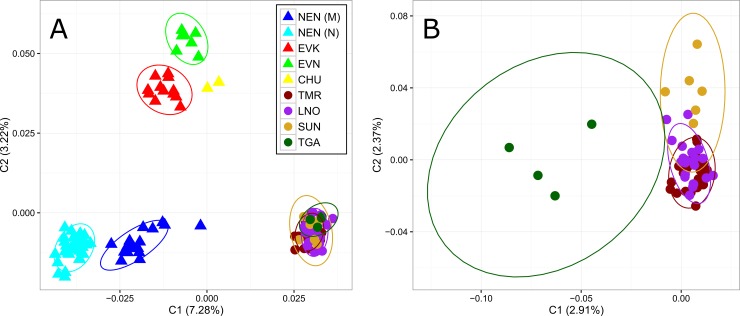
**Multidimensional scaling analysis of reindeer populations: A–wild and domestic reindeer populations based on 8357 SNPs and B–wild population based on 8145 SNPs**. Note: NEN_M–Nenets breed of the Murmansk region; NEN_N–Nenets breed of the Nenets Autonomous district; EVK–Evenk breed; EVN–Even breed; CHU–Chukotka-Khargin breed; and TMR–Taymyr Peninsula, LNO–Leno-Olenek, SUN–Sundrun, and TGA–taiga (boreal forest) wild populations.

The level of similarity of individual relationships within and among genotyped reindeer populations was visualized with MDS (Fig 3).

MDS revealed a clear differentiation of wild and domestic populations along axis 1 (Fig 3A). Component 1 (C1) accounted for 7.28% of the variability and showed that NEN_M varied from other domestic breeds. Component 2 (C2) accounted for 3.22% of the variance and discriminated the remaining breeds of domestic reindeer. In the MDS plot constructed for wild reindeer (Fig 3B), C1 explained 2.91% of the variability and split the TGA population from the others. C2 accounted for 2.37% of variability and separated the SUN population, while TMR and LNO populations formed partly overlapping clusters.

### Genetic structure analysis

**Fig 4:**
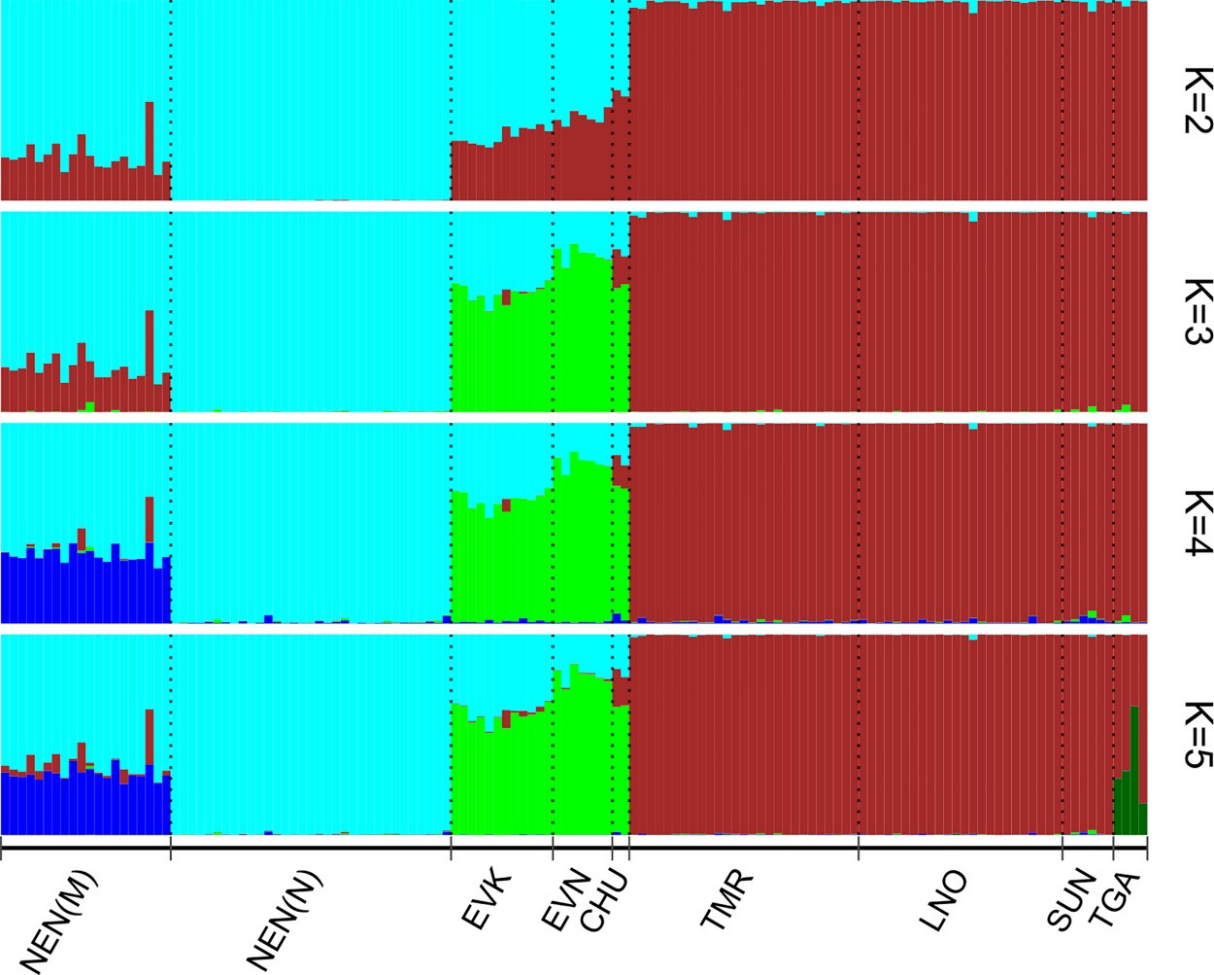
Population structure analysis of reindeer populations inferred using STRUCTURE software based on 8357 SNPs for K values from 2 to 5. Note: NEN_M–Nenets breed of the Murmansk region; NEN_N–Nenets breed of the Nenets Autonomous district; EVK–Evenk breed; EVN–Even breed; CHU–Chukotka-Khargin breed; and TMR–Taymyr Peninsula, LNO–the Leno-Olenek, SUN–Sundrun, and TGA–taiga (boreal forest) wild populations.

The results of STRUCTURE analysis from K = 2 to K = 5 are shown in Fig 4. Although the most probable Δ K value was found at K = 2 and the second largest ΔK at K = 4 (S1 Fig), we presented the results for K = 5 due to their relevance.

At K = 2, the main structure of domestic and wild populations was revealed. Among domestic forms, only NEN_N was clearly differentiated from their wild relatives. We observed strong admixture signals from the wild reindeer in other groups of domestic reindeer. At K = 3, the wild-genomic component was maintained in NEN_M and CHU. At higher K-values, the wild-specific component was still visible in CHU and partly NEN_M. The different origin of TGA was revealed in a specific pattern at K = 5. Among domestic reindeer, the NEN_N appeared to be the most genetically pure, and other breeds had admixed patterns with significant NEN_N-specific components.

### TreeMix

**Fig 5:**
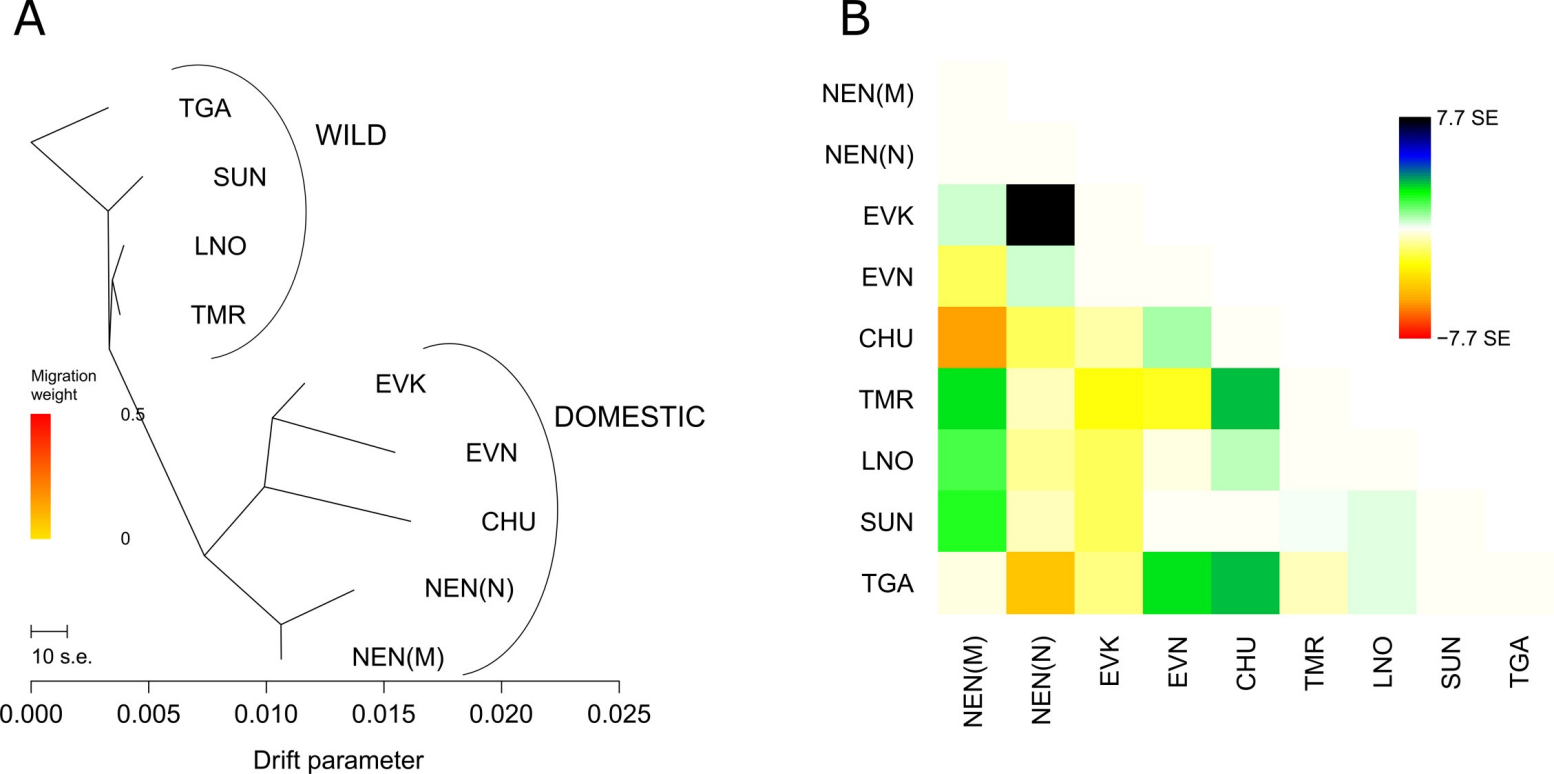
**A. Maximum likelihood tree of all studied reindeer groups with no migration events based on 8357 SNPs. B. The residual plot, corresponding to the tree with no migration events based on 8357 SNPs.** Note: NEN_M–Nenets breed of the Murmansk region; NEN_N–Nenets breed of the Nenets Autonomous district; EVK–Evenk breed, EVN–Even breed; CHU–Chukotka-Khargin breed; TMR–Taymyr Peninsula, LNO–Leno-Olenek, SUN–Sundrun, and TGA–taiga (boreal forest) wild populations. Colors are described in the palette on the right.

To determine the patterns of the population splits and gene flow in the studied reindeer groups, we further applied the TreeMix approach [48]. TGA was set as a root out-group. We constructed a maximum likelihood tree with no migration events (Fig 5A), in which wild and domestic populations were divided by their own clusters. In addition, the tree placed all regional domestic populations of Yakutia (EVN, EVK, and CHU) adjacent to each other and distanced them from the Nenets breeds (NEN_N and NEN_M).

The residual plot, corresponding to the tree, showed a more detailed arrangement of the groups (Fig 5B). A positive residual suggested that EVK and NEN_N were more closely related to each other, while a negative residual indicated that TGA and SUN were less closely related to each other in the data than in the best-fit tree.

**Fig 6:**
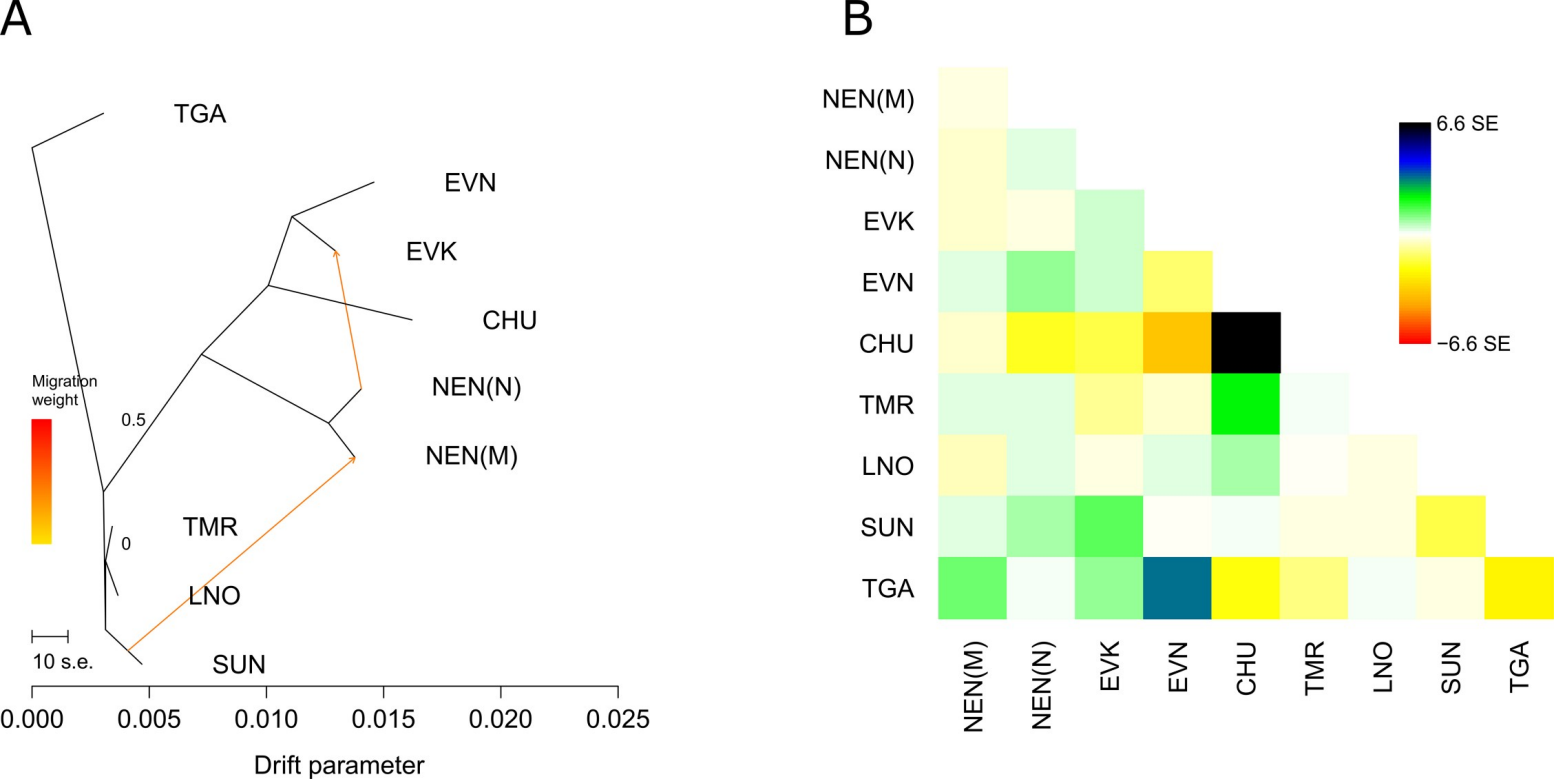
**A. Maximum likelihood tree of all studied reindeer groups with two migration events based on 8357 SNPs. B. The residual plot, corresponding to the tree with two migration events based on 8357 SNPs.** Note: NEN_M–Nenets breed of the Murmansk region; NEN_N–Nenets breed of the Nenets Autonomous district; EVK–Evenk breed, EVN–Even breed; CHU–Chukotka-Khargin breed; TMR–Taymyr Peninsula, LNO–Leno-Olenek, SUN–Sundrun, and TGA–taiga (boreal forest) wild populations. Colors are described in the palette on the right.

Then, we sequentially increased the migration edges. Notable results were observed when two migration events were allowed in the tree model (p<0.05 for all reported migration edges). (Fig 6A).

Two significant mixture events were found: from the branch of the wild populations to the NEN_M and from NEN_N to EVK. The corresponding residual (Fig 6B) for this tree suggested migration events between EVN and TGA.

For a better understanding of population relationships in the studied reindeer groups, we applied the *f*_3_ statistic, which is defined as the product of allele frequency differences between population C to A and B [49]. This test examines if a target population C is admixed between two source populations (A and B). Although the obtained product can be positive or negative, an evidence of admixture in the history of population C is confirmed only by a significantly negative value.

Significantly negative Z-scores (Table 3) were observed in the combination of NEN_M with NEN_N and all wild reindeer groups, indicating that NEN_M originated from an admixture event from both domestic and wild populations.

**Table 3:** Significantly negative Z-scores of the *f*_3_ statistic in 10 blocks.

Target population	Source 1	Source 2	*f*_3__statistics	Standard error	Z-Score	P-value
NEN_M	NEN_N	TMR	-0.000563707	0.000141592	-3.9812	6.856822e-05
NEN_M	NEN_N	LNO	-0.000517611	0.000142631	-3.62902	0.0002844992
NEN_M	NEN_N	SUN	-0.000706648	0.000172375	-4.09948	4.140794e-05
NEN_M	NEN_N	TGA	-0.000643368	0.000198698	-3.23792	0.001204046

Note: NEN_M–Nenets breed of the Murmansk region; NEN_N–Nenets breed of the Nenets Autonomous district; TMR–Taymyr Peninsula, LNO–Leno-Olenek, SUN–Sundrun, and TGA–taiga (boreal forest) wild populations.

## Discussion

Commercially developed livestock genotyping arrays have been used in several studies to identify novel SNPs in closely evolutionarily related non-model species [50, 51], including those for the family Cervidae [30, 31; 33]. In our study, a total of 135 domestic and wild reindeer, inhabiting the Nenets Autonomous district, Murmansk region, western Taymyr, and Yakutia, were genotyped with Illumina BovineHD BeadChip. Of the total 777,962 SNPs on the BovineHD array, 8357 SNP markers were polymorphic and were used to assess the genetic variability and population structure of the studied reindeer populations. In comparison, Kasarda et al. [31], using the BovineSNP50 BeadChip, identified 1542 polymorphic SNPs and estimated the genetic diversity of farmed and free-range red deer (*Cervus elaphus*) and fallow deer (*Dama dama*) was estimated. Haynes and Latch [30], after applying the same genotyping array to *Odocoileus hemionus* (mule deer and black-tailed deer) and *O*. *virginianus* (white-tailed deer) in the Pacific Northwest (USA), found that 38.7% of loci could be genotyped, of which 5% (n = 1068) were polymorphic. Additionally, the authors showed that the three types of deer could readily be distinguished with this SNP dataset. The first whole-genome analysis of Russian reindeer was conducted using two sets of commercially available SNP chips developed for cattle (BovineSNP50 BeadChip) and sheep (OvineSNP50 BeadChip) [33]. This application revealed the call rates for fully scored SNP at 43.0 and 47.0%, respectively, and 5.3% (1257 SNPs) and 2.0% (519 SNPs) of them were found to be polymorphic. The lower rate of genotyping success in these studies, including the current work, is expected, given the 25.1−30.1-million-year divergence between Bovidae (*Bos taurus*) and Cervidae (*O*. *hemionus* and *O*. *virginianus*). However, since the initial genotyping array had a sufficient number of loci, even a low proportion of cross-amplifying SNPs represents a useful set of markers for species that lack genomic resources [30].

Genetic diversity is the key pillar of biodiversity and diversity within species, between species, and of ecosystems [52]. Reindeer are an essential element of Russia’s Far North ecosystem. Due to their value, reindeer diversity across populations has long fascinated scientists. The first results were obtained in the 1960s and wide application of the gel electrophoresis method followed [53–56]. Further advent of more subtle methods, like mitochondrial [4; 14; 57–59] and microsatellite [4; 60–64] markers, allowed researchers to determine the phylogeny and origin of reindeer individuals, as well as to estimate the genetic variability and degree of differentiation between populations. An application of a commercial BovineSNP50K BeadChip revealed a generally high level of diversity in Russian reindeer [23; 34], which was confirmed by our present research. The analysis of all calculated parameters revealed a tendency toward higher genetic variability in wild populations, compared to domestic ones (Table 1). Similarly, a higher level of genetic diversity in wild reindeer populations was detected using BovineSNP50 v2 BeadChip [34]. Both populations demonstrated a deficiency of heterozygotes with its prevalence in domestic reindeer, *F*_IS_ = 0.026, compared to *F*_IS_ = 0.044 in wild reindeer. This can be explained by the fact that domestic reindeer are isolated due to husbandry, while wild reindeer make long migrations and have more opportunities to exchange genetic material, which contributes to conserving biodiversity. Among domestic reindeer, the highest level of genetic variability was detected in EVK (*H*o = 0.172, *H*e = 0.175, *A*r = 1.319). Calculation of the genetic diversity observed within wild reindeer groups followed similar trends, except for TGA. The small census size and geographical isolation resulted in inbreeding in this population, and consequently reduced genetic variability.

From our analyses, the pairwise *F*_ST_ values for domestic and wild reindeer showed high genetic differentiation between each population and all *F*_ST_ values were significantly different from 0 (p<0.01) (Table 2). Likewise, the high levels of pairwise genetic differences among regional populations of domestic and wild reindeer across northeastern Zabaĭkal'e was evident from the microsatellite markers [4]. We further revealed that the population tree based on pairwise *F*_*ST*_ values (Fig 2), an individual MDS plot based on pairwise identity-by-state distance matrix (Fig 3A), and the maximum likelihood tree with no migration events (Fig 5A) showed a high degree of divergence between the four regional populations of domestic reindeer and TMR and TGA. An interesting pattern was observed in the MDS plot for both populations: each domestic group formed its own cluster, although all wild individuals were characterized by significant overlapping wild-specific clusters. The notably more distinct genetic structure of domestic populations could be explained by the influence of anthropogenic factors (artificial selection for certain traits). According to Syroechkovskii [17], domestic reindeer are characterized by several features that distinguish them from wild reindeer. The domestic form is more accustomed to eating lichens, which is a characteristic developed during in the process of domestication. The diet of wild individuals is much more diverse. Domestic animals tend to be slightly smaller than wild reindeer and have slightly different color patterns, but they are remarkably similar in appearance [65]. Highly trained domestic reindeer are mainly used for milking and transport and are rarely slaughtered for food. This restriction does not apply to the wild reindeer. Local herdsmen usually establish camps near groups of wild forest reindeer, which are an important source of food and skins for clothing and equipment [4]. The domestic population is characterized by a very strong and constant instinct of herding, which in the wild is poorly developed and strictly limited to migration [66]. The strong differentiation between wild and domestic reindeer was also confirmed by STRUCTURE analysis (Fig 4). At K = 2, the assignment of each population to its own cluster was revealed, although some signs of admixture were detected in the domestic regional populations. Furthermore, some admixture was obtained between the Even breed and the wild taiga population from the residual plot corresponding to the tree with two migration events (Fig 6B). This could be explained by reindeer moving from the taiga to the tundra. Indeed, a close genetic and morphological similarity between the wild forest reindeer and domestic reindeer in Yakutia has been validated [20].

The genetic differences between wild and domestic reindeer have also been reported by scientists from different countries. Jepsen et al. [67], using the nuclear microsatellite, revealed distinct differences between caribou and domestic reindeer in southwest Greenland. Due to few caribou in Greenland, Norwegian semi-domestic reindeer were introduced in 1952. A likely explanation for the genetic isolation of the investigated populations is that natural barriers (glaciers and wide fjords) exist in the area. The genetic relationships between reindeer and caribou in Alaska were investigated by Cronin et al. [55]. Reindeer were introduced into Alaska 100 years ago and have been maintained as semi-domestic livestock. They have had contact with wild caribou herds, including deliberate crossbreeding and mixing in the wild. Authors identified most alleles in both reindeer and caribou, which may be the result of recent common ancestry or genetic introgression in either direction. Regarding the genetic diversity of migrating caribou herds on the Alaska North Slope and their potential hybridization with introduced domestic reindeer, Mager et al. [68] detected several individuals of mixed genetic origin (8% in caribou populations and 14% among domestic reindeer).

The wild and domestic populations in Russia differ from those mentioned above: they have coexisted for centuries. Our study based on genotyping data obtained with a chip designed for dairy and beef cattle breeds (BovineHD BeadChip), revealed a clear and high genetic differentiation between domestic and wild reindeer inhabiting the vast territory of the Russian Far North, an area extending over 4000 km from the west to the east.

Further research has also focused on differences between domestic and wild reindeer forms. In the late 1970s, scholars such as Zabrodin et al. [69] concluded that the morphological differences between various domestic reindeer populations in northern Russia were not sufficiently significant to constitute distinct breeds. Nevertheless, later Russian publications agreed on the existence of four breeds [8; 70–72]. Therefore, in 1985, the Russian Ministry of Agriculture (formerly the USSR Ministry of Agriculture) officially recognized four reindeer breeds: Even, Evenk, Chukotka, and Nenets. In addition, due to natural and economic conditions and specific feeding and selective-breeding systems, several within-breed ecotypes developed in different areas [73]. For instance, the Khargin reindeer are raised in Yakutia, though they are of Chukotka origin. The Chukchi exclusively hunted wild reindeer as late as the turn of the 18th century, whereupon they acquired reindeer husbandry from the Koryaks. After they turned to reindeer husbandry, they moved to Yakutia with their reindeer that were different in conformation, feeding habits, and behavior from that of their Koryak ancestors. In Yakutia, the Khargin reindeer found themselves alongside Evenki reindeer that were raised in the forests of the Okhotsk Sea coast and differed from their Chukotka conspecifics [70]. However, the Nenets is the most geographically diverse b breed. These ecotypes markedly differ in terms of appearance and body measurements, and the largest breed is raised in the Murmansk region.

Reindeer breeds are not only morphological and ecologically distinct but are also genetically different. Thus, significant differences between some breeds were detected by studying transferrin locus polymorphism [66], microsatellite variability [74], and SNPs with a medium-density DNA chip [23; 34].

Our genetic analyses, using Neighbor-Net tree based on pairwise *F*_*ST*_ (Fig 2) and MDS analysis (Fig 3A), revealed that each domestic regional population formed its own cluster and was placed on the plot consistent with its known geographical origin. Namely, the breeds raised in Yakutia had neighboring locations, and the two populations of the Nenets breed formed their own branch. Interestingly, the MDS plot of the same Yakutia populations genotyped with BovineSNP50 BeadChip showed minor overlapping with the Even and Evenk clusters [23]. Presumably, the polymorphic loci obtained in that study (512 SNPs) were not sufficient for clear differentiation of the breeds. Nevertheless, we noticed that the second MDS component clearly distinguished the populations of the Nenets breed and the wild group from the rest of the domestic regional populations. Furthermore, NEN_M was placed between NEN_N and the wild group. Likewise, although the Structure analysis (Fig 4) clearly differentiated domestic populations in accordance with their geographic location, NEN_M represented an admixture pattern with NEN_N and with a greater part of an unknown population. The Murmansk region (the Kola Peninsula) was the area of the expansion of the Saami people who bred the Saami-type reindeer, which were later replaced by the Nenets breed [75]. The displacement of the Saami by the Nenets continued until 1887, when the latter came with their herds from Pechora to the Kola Peninsula and began to supplant the local reindeer herders from their best pastures [76]. Thus, by 1928, the Saami owned less than half (46%) of the peninsula reindeer population [77]. Based on these findings, we hypothesize that NEN_M contains alleles that were inherited from the Saami reindeer and NEN_N. This conclusion was confirmed by the results of TreeMix and *f*_3_ statistic. The maximum likelihood tree for two migration events (Fig 6A) identified significant gene flow from the wild group to NEN_M. A significant negative *f*_3_ statistic (Table 3) showed that NEN_M originated from two ancestral populations: NEN_N and wild. Additionally, the residual plot for the corresponding tree with no migration events (Fig 5B) revealed that EVK and NEN_N were genetically closer than they were presented in the tree. Natural gene flow over such a distance could not be possible, and one putative explanation could be the influence of human actions. Occurrences of reindeer exchange between regions are very well known [8; 66]. However, evidence of animal exchange between the Nenets Autonomous district and Yakutia has not been observed.

The genetic differentiation of wild reindeer has been widely studied using different molecular markers. Thus, heterogeneity transferrin locus was detected within western and eastern groups of the Taymyr population [56; 78]. This pattern was later confirmed by the analysis of the mtDNA control region [3]. Phylogenetic analysis based on mtDNA sequences of reindeer from the continental part of the northeast Russia showed a close relationship with animals from the Siberian tundra [59]. Using 16 microsatellite loci, Baranova et al. [79] found poor separation of reindeer from eastern Eurasia in some habitats (Tomsk region, Khanty-Mansy Autonomous district, Taymyr, Yakutia, and Chukotka), as evidenced by their close genetic relationship. Wild reindeer in Russia include tundra and taiga herds. Among tundra reindeer, the Taymyr population is the largest in the world [80]. Likewise, three large herds of tundra reindeer inhabit Taymyr and Yakutia: Lena-Olenek, Yana-Indigirka, and Sundrun [70]. Forest reindeer were divided into Siberian and Okhotsk [81]. However, several authors inferred from their studies that the forest reindeer of Evenkia, Trans-Baikal Territory, Southern Yakutia, and Far East were the same subspecies [82–84]. Based on 8145 SNPs, we investigated the population structure and genetic differentiation within the Taymyr, Lena-Olenek, Sundrun, and wild taiga reindeer using several approaches. The lowest values of *F*_*ST*_ were observed between groups of the tundra form (TMR/LNO = 0.004, TMR/SUN = 0.010, and LNO/SUN = 0.009) while the wild taiga population was equally genetically distant from them (TGA/TMR = 0.034, TGA/LNO = 0.031, and TGA/SUN = 0.035) (Table 1). Furthermore, close relationships between TMR, LNO, and SUN were observed using Neighbor-Net tree (Fig 2), MDS (Fig 3B), and a maximum likelihood tree (Figs 5A and 6A). We observed that TMR and LNO populations were very tightly clustered. This could be due to documented Taymyr population migrations to northwest Yakutia. The first observed migrations of the Taimyr population were noted in the 1980s [17], they became more regular and grew in number at the beginning of the 21st century. Due to many factors, including human activity, this population began to change traditional migrations [85]. Krivoshapkin [19] estimated the total number of the migrating Taymyr herds traveling to northwest Yakutia in October 2013 at 22–24,000 individuals. The STRUCTURE results from K = 2 to K = 4 revealed that all wild reindeer formed their own cluster (Fig 4). However, at K = 5, the forest reindeer tended to have an independent cluster with a proportion of tundra individuals. Sharp distinctions between tundra and forest reindeer ecology and behavior have been described [8; 17; 70]. Forest reindeer have a darker coat and smaller antlers than their tundra conspecifics. According to Egorov [86], these features are particularly useful in the forest. Tundra reindeer migrate great distances over routes largely determined not by terrain landmarks, but by availability of forage, changes in snow cover, dates of river freezing and breakup, climate, and the impact of bloodsucking insects [70].

## Conclusions

To our knowledge, this study is the first of its kind aiming to investigate the genetic diversity, structure, and differentiation of four domestic regional populations and two wild reindeer populations of western Taymyr and the taiga and tundra zones of Yakutia, using a genome-wide bovine genotyping array. Using different population genetics approaches, our research yielded valuable results: 1) the existence of strong genetic population structure and differentiation between domestic and wild reindeer populations of the Russian Far North; 2) wild reindeer were characterized by higher genetic diversity; 3) each domestic regional population showed a distinct genetic structure, although two of these represented an admixture pattern with the wild population; and 4) differences in morphological and ecological features of tundra and taiga reindeer were confirmed by differences and contrasting patterns in genetic structures.

Thus, our study provides novel insights into the genetic diversity and population structure of reindeer, supporting further resource utilization and development of genetic improvement strategies and conservation programs for this species.

## Supporting information

S1 Fig Delta K plot showing a peak at K = 2 and K = 4. (TIFF)
