# Supplementary figures and images for: Genetic diversity and population structure of domestic and wild reindeer (Rangifer tarandus L. 1758): A novel approach using BovineHD BeadChip
Source: PLoS One. 2018 Nov 30;13(11):e0207944. doi: 10.1371/journal.pone.0207944 (PMC6267972; doi:10.1371/journal.pone.0207944)

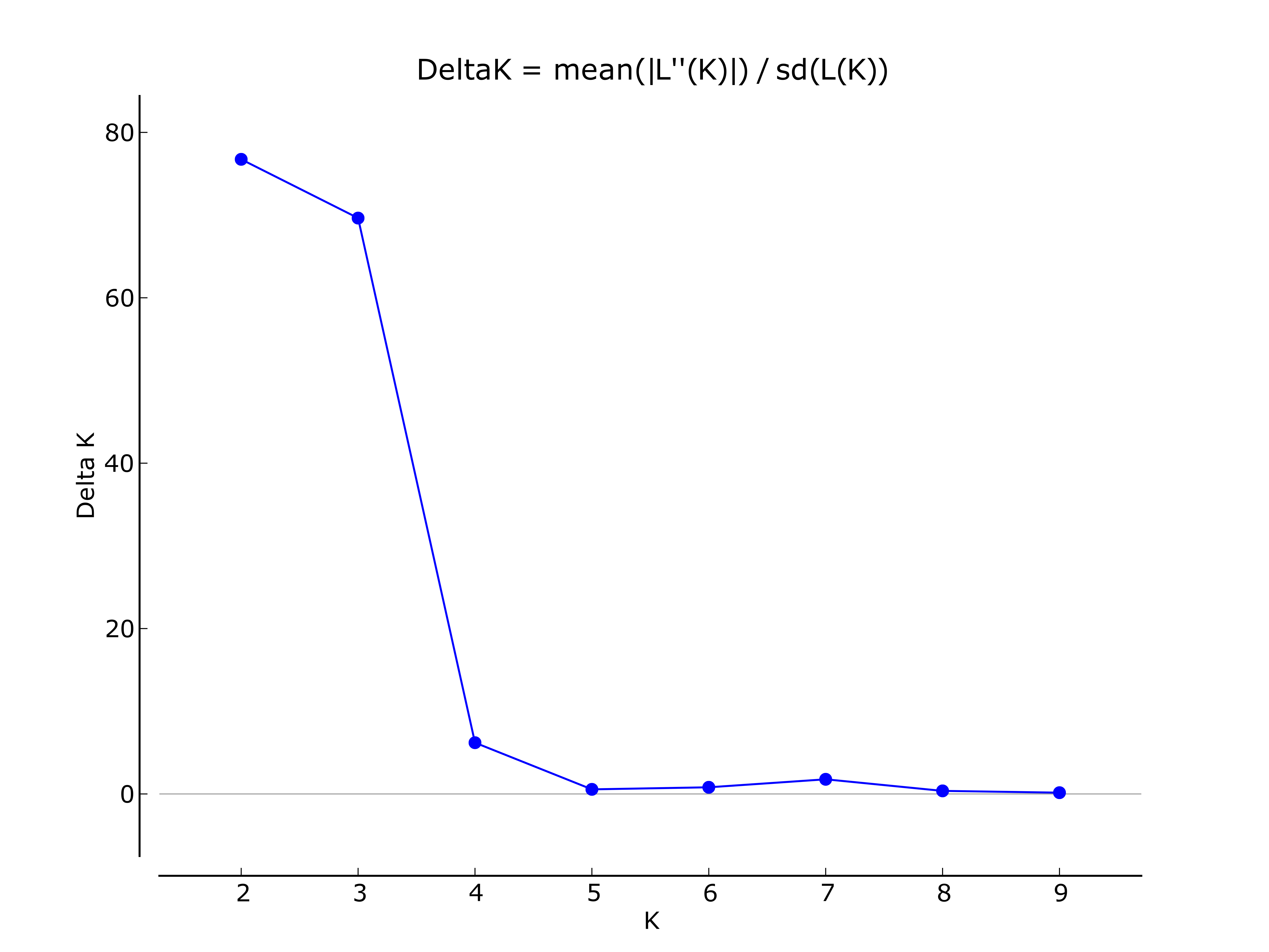

Supplement: S1 Fig — (TIFF) [file pone.0207944.s001.tiff]
